# Supplementary material for: Epidemiology and treatment outcomes of recurrent tuberculosis in Tanzania from 2018 to 2021 using the National TB dataset
Source: PLoS Negl Trop Dis. 2024 Feb 15;18(2):e0011968. doi: 10.1371/journal.pntd.0011968 (PMC10901333; doi:10.1371/journal.pntd.0011968)
Supplement: S1 Table — (DOCX) [file pntd.0011968.s001.docx]

**Supplementary Table 1: Bivariate analysis of factors associated with TB recurrence in Tanzania from 2018 to 2021**

| **Variables** | **TB Recurrence** | | | | **Total** | **p value** |
| --- | --- | --- | --- | --- | --- | --- |
|  | **No** | | **Yes** | |  |  |
|  | **n** | **%** | **n** | **%** | **n** |  |
| **Age group (years)** |  |  |  |  |  | <0.001 |
| 0-14 | 48,617 | 99.5 | 261 | 0.5 | 48,878 |  |
| 15-24 | 28,250 | 98.8 | 357 | 1.2 | 28,607 |  |
| 25-49 | 137,839 | 97.6 | 3,354 | 2.4 | 141,193 |  |
| 50+ | 98,701 | 97.7 | 2,338 | 2.3 | 101,039 |  |
| **Sex** |  |  |  |  |  | <0.001 |
| Female | 124,836 | 98.5 | 1,896 | 1.5 | 126,732 |  |
| Male | 188,572 | 97.7 | 4,414 | 2.3 | 192,986 |  |
| **HIV status^1^** |  |  |  |  |  | <0.001 |
| Negative | 240,866 | 98.3 | 4,266 | 1.7 | 245,132 |  |
| Positive | 70,402 | 97.2 | 1,994 | 2.8 | 72,396 |  |
| **TB type** |  |  |  |  |  | <0.001 |
| Both | 768 | 98.5 | 12 | 1.5 | 780 |  |
| Extra pulmonary | 64,420 | 98.9 | 714 | 1.1 | 65,134 |  |
| Pulmonary | 248,107 | 97.8 | 5,583 | 2.2 | 253,690 |  |
| **TB referral types** |  |  |  |  |  | <0.001 |
| CTC | 32,004 | 97.0 | 1,006 | 3.0 | 33,010 |  |
| Community | 87,063 | 98.3 | 1,523 | 1.7 | 88,586 |  |
| Self-referrals | 167,703 | 98.1 | 3,293 | 1.9 | 170,996 |  |
| Others^2^ | 26,637 | 98.2 | 488 | 1.8 | 27,125 |  |
| **Facility level** |  |  |  |  |  | <0.001 |
| Dispensary | 79,315 | 98.2 | 1,428 | 1.8 | 80,743 |  |
| Health Centre | 89,486 | 98.0 | 1,838 | 2.0 | 91,324 |  |
| Hospitals | 144,609 | 98.0 | 3,044 | 2.0 | 147,653 |  |
| **Geographical zones^3^** |  |  |  |  |  | <0.001 |
| Central | 39,321 | 98.1 | 770 | 1.9 | 40,091 |  |
| Coastal | 91,092 | 97.3 | 2,485 | 2.7 | 93,577 |  |
| Lake | 77,070 | 98.3 | 1,332 | 1.7 | 78,402 |  |
| Northern | 54,158 | 98.4 | 887 | 1.6 | 55,045 |  |
| Southern Highlands | 41,029 | 98.6 | 565 | 1.4 | 41,594 |  |
| Western | 7,834 | 97.6 | 191 | 2.4 | 8,025 |  |
| Zanzibar | 2,906 | 97.3 | 80 | 2.7 | 2,986 |  |
| **DOT option** |  |  |  |  |  | <0.001 |
| Facility | 4,445 | 80.9 | 1,047 | 19.1 | 5,492 |  |
| Community | 297,551 | 98.4 | 4,925 | 1.6 | 302,476 |  |
| **TB diagnostic method^4^** |  |  |  |  |  | <0.001 |
| Bacteriologically confirmed | 202,688 | 97.6 | 5,046 | 2.4 | 207,734 |  |
| Clinically diagnosed | 77,082 | 98.6 | 1,061 | 1.4 | 78,143 |  |
| **TB treatment regimen** |  |  |  |  |  | <0.001 |
| 2HRZE/10RH^5^ | 3,932 | 97.1 | 117 | 2.9 | 4,491 |  |
| 2RHZE/4RH^6^ | 305,161 | 98.2 | 5,652 | 1.8 | 310,813 |  |
| 2SRHZE/1RHZE/5RHE or 3RHZE/5RHE^7^ | 232 | 50.2 | 230 | 49.8 | 462 |  |
| **Year of TB diagnosis** |  |  |  |  |  | <0.001 |
| 2018 | 73,600 | 97.6 | 1,814 | 2.4 | 75,414 |  |
| 2019 | 80,399 | 98.0 | 1,657 | 2.0 | 82,056 |  |
| 2020 | 80,892 | 98.0 | 1,630 | 2.0 | 82,522 |  |
| 2021 | 78,519 | 98.5 | 1,209 | 1.5 | 79,728 |  |

CTC: Centre for Treatment and Care; DS-TB: Drug-sensitive TB; DOT: Directly Observed Therapy; TB: Tuberculosis; HRZE: Isoniazid, Rifampicin, Pyrazinamide, Ethambutol; SRHZE: Streptomycin, Rifampicin, Isoniazid, Pyrazinamide, Ethambutol. ^1^Patients with unknown HIV status excluded from analysis. ^2^Patients referred from inpatient department (IPD), outpatient department (OPD), diabetic clinic, voluntary counselling and testing (VCT), reproductive and child health clinics and others not defined. ^3^Countries included in the geographical zones: Northern zone: Kilimanjaro, Tanga, Arusha, and Manyara. Coastal zone; Morogoro, Dar es Salaam, Pwani, Lindi, and Mtwara. Western zone: Katavi and Kigoma. Central zone: Tabora, Dodoma, and Singida. Lake zone: Kagera, Mwanza, Geita, Mara, Simiyu, and Shinyanga. Southern highlands zone: Songwe, Ruvuma, Mbeya, Njombe, Rukwa and Iringa. Zanzibar: Pemba and Unguja. ^4^TB diagnosis method: Bacteriologically confirmed diagnostic method: Gene Xpert, Microscopy and culture. Clinical diagnostic method: TB chart card and Chest X-ray. ^5^First line treatment regimen for extra-pulmonary TB. ^6^First line treatment regimen for pulmonary TB. ^7^Previous TB treatment regimen for patients with relapse TB or “Other” TB category before the new TB retreatment regimen introduced in 2019
