# Supplementary material for: Epidemiology and treatment outcomes of recurrent tuberculosis in Tanzania from 2018 to 2021 using the National TB dataset
Source: PLoS Negl Trop Dis. 2024 Feb 15;18(2):e0011968. doi: 10.1371/journal.pntd.0011968 (PMC10901333; doi:10.1371/journal.pntd.0011968)
Supplement: S2 Table — (DOCX) [file pntd.0011968.s002.docx]

**Supplementary Table 2: Bivariate analysis of factors associated with unfavourable treatment outcomes among patients with recurrent TB in Tanzania from January 2018 to December 2021**

| **Variables** | **Treatment Outcome** | | | | **Total** | ***p value*** |
| --- | --- | --- | --- | --- | --- | --- |
|  | **Favourable outcomes** | | **Unfavourable outcomes** | |  |  |
|  | **n** | **%** | **n** | **%** | **n** |  |
| **Age group (years)** |  |  |  |  |  | 0.142 |
| 0-14 | 224 | 94.1 | 14 | 5.9 | 238 |  |
| 15-24 | 291 | 89.3 | 35 | 10.7 | 326 |  |
| 25-49 | 2,595 | 89.9 | 290 | 10.1 | 2,885 |  |
| 50+ | 1,790 | 90.9 | 180 | 9.1 | 1,970 |  |
| **Sex** |  |  |  |  |  | 0.672 |
| Female | 1,448 | 90.2 | 158 | 9.8 | 1,606 |  |
| Male | 3,452 | 90.5 | 361 | 9.5 | 3,813 |  |
| **HIV status^1^** |  |  |  |  |  | <0.001 |
| Negative | 3,345 | 92.1 | 288 | 7.9 | 3,633 |  |
| Positive | 1,533 | 87.1 | 228 | 12.9 | 1,761 |  |
| **TB type** |  |  |  |  |  |  |
| Both | 8 | 100.0 | 0 | 0.0 | 8 | 0.208 |
| Extra pulmonary | 550 | 92.1 | 47 | 7.9 | 597 |  |
| Pulmonary | 4,341 | 90.2 | 472 | 9.8 | 4,813 |  |
| **TB related referrals** |  |  |  |  |  | <0.001 |
| CTC | 775 | 87.1 | 115 | 12.9 | 890 |  |
| Community | 1,090 | 90.0 | 121 | 10.0 | 1,211 |  |
| Self-referrals | 2,679 | 91.8 | 238 | 8.2 | 2,917 |  |
| Others^2^ | 356 | 88.8 | 45 | 11.2 | 401 |  |
| **Facility level** |  |  |  |  |  | 0.115 |
| Dispensary | 1,111 | 91.5 | 103 | 8.5 | 1,214 |  |
| Health Centre | 1,420 | 89.3 | 171 | 10.7 | 1,591 |  |
| Hospitals | 2,369 | 90.6 | 245 | 9.4 | 2,614 |  |
| **Geographical zones^3^** |  |  |  |  |  | <0.001 |
| Central | 621 | 89.6 | 72 | 10.4 | 693 |  |
| Coastal | 2,000 | 88.5 | 261 | 11.5 | 2,261 |  |
| Lake | 958 | 92.1 | 82 | 7.9 | 1,040 |  |
| Northern | 678 | 94.0 | 43 | 6.0 | 721 |  |
| Southern Highlands | 425 | 91.4 | 40 | 8.6 | 465 |  |
| Western | 150 | 92.6 | 12 | 7.4 | 162 |  |
| Zanzibar | 68 | 88.3 | 9 | 11.7 | 77 |  |
| **DOT option** |  |  |  |  |  | <0.001 |
| Community | 3,882 | 91.8 | 347 | 8.2 | 4,229 |  |
| Facility | 844 | 87.4 | 122 | 12.6 | 966 |  |
| **TB diagnostic method^4^** |  |  |  |  |  | 0.002 |
| Bacteriologically confirmed | 3,867 | 89.8 | 437 | 10.2 | 4,304 |  |
| Clinically diagnosed | 896 | 93.1 | 66 | 6.9 | 962 |  |
| **TB treatment regimen** |  |  |  |  |  | 0.007 |
| 2HRZE/10RH^5^ | 54 | 88.5 | 7 | 11.5 | 61 |  |
| 2RHZE/4RH^6^ | 4,494 | 91.2 | 431 | 8.8 | 4,925 |  |
| 2SRHZE/1RHZE/5RHE or 3RHZE/5RHE^7^ | 190 | 85.2 | 33 | 14.8 | 223 |  |
| **Year of TB diagnosis** |  |  |  |  |  | 0.094 |
| 2018 | 1,578 | 89.2 | 192 | 10.8 | 1,770 |  |
| 2019 | 1,463 | 91.2 | 141 | 8.8 | 1,604 |  |
| 2020 | 1,442 | 91.3 | 137 | 8.7 | 1,579 |  |
| 2021 | 417 | 89.5 | 49 | 10.5 | 466 |  |

CTC: Centre for Treatment and Care; DS-TB: Drug-sensitive TB; DOT: Directly Observed Therapy; TB: Tuberculosis; HRZE: Isoniazid, Rifampicin, Pyrazinamide, Ethambutol; SRHZE: Streptomycin, Rifampicin, Isoniazid, Pyrazinamide, Ethambutol. ^1^Patients with unknown HIV status excluded from analysis. ^2^Patients referred from inpatient department (IPD), outpatient department (OPD), diabetic clinic, voluntary counselling and testing (VCT), reproductive and child health clinics and others not defined. ^3^Countries included in the geographical zones: Northern zone: Kilimanjaro, Tanga, Arusha, and Manyara. Coastal zone; Morogoro, Dar es Salaam, Pwani, Lindi, and Mtwara. Western zone: Katavi and Kigoma. Central zone: Tabora, Dodoma, and Singida. Lake zone: Kagera, Mwanza, Geita, Mara, Simiyu, and Shinyanga. Southern highlands zone: Songwe, Ruvuma, Mbeya, Njombe, Rukwa and Iringa. Zanzibar: Pemba and Unguja. ^4^TB diagnosis method: Bacteriologically confirmed diagnostic method: Gene Xpert, Microscopy and culture. Clinical diagnostic method: TB chart card and Chest X-ray. ^5^First line treatment regimen for extra-pulmonary TB. ^6^First line treatment regimen for pulmonary TB. ^7^Previous TB treatment regimen for patients with relapse TB or “Other” TB category before the new TB retreatment regimen introduced in 2019
