# Supplementary material for: Epidemiology and treatment outcomes of recurrent tuberculosis in Tanzania from 2018 to 2021 using the National TB dataset
Source: PLoS Negl Trop Dis. 2024 Feb 15;18(2):e0011968. doi: 10.1371/journal.pntd.0011968 (PMC10901333; doi:10.1371/journal.pntd.0011968)
Supplement: Attachment — Submitted filename: Responses to reviewer_04012024.docx [file pntd.0011968.s003.docx]

**04^th^ January 2024**

Dear Editor and Editorial Team,

PLOS Neglected Tropical Diseases

**RE: Submission of a revised manuscript for publication in PLOS Neglected Tropical Diseases Journal**

Thank you very much for the reviews and the opportunity to revise the manuscript. We have carefully revised the manuscript and will upload clean and tracked changed versions. In addition, we have attached a document with detailed responses to the comments and suggestions.

On behalf of all the authors of the above-noted manuscript, I confirm that this submission has not been published and is not being considered for publication elsewhere and that all the authors have agreed to the submission of this manuscript to PLOS Neglected Tropical Diseases Journal.

Sincerely,

Belinda J Njiro

(On behalf of all co-authors)

**Responses to Reviewers’ comments**

Comments -Reviewer #1: The objective was clearly articulated in the hypothesis test, the study was designed well to address the objective and the population was clearly described with the test. The sample size sufficiently ensured the hypothesis and the analysis with good statistical support. the study was concerned with ethically approved

**Response**: We thank the reviewer for this comment.

Comments -Reviewer #2: The authors set out to conduct a cross-sectional study of the determinants of treatment outcomes in recurrent TB. The hypothesis, results, and statistical tests are represented clearly and tested appropriately. They have met the ethical and regulatory requirements.

**Response**: We thank the reviewer for this comment.

Comments -Reviewer #3: Interesting study with clear and articulate objectives and hypothesis. But the study design is NOT a cross-sectional study but rather a retrospective cohort study with a secondary data analysis. The study population or participants for the study is not clear; is it general TB patients or TB recurrence patients? Looking at the title of the study. This lack of clarity is seen in the analysis outputs.

**Responses:**

Comment 1:

But the study design is NOT a cross-sectional study but rather a retrospective cohort study with a secondary data analysis.

**Response:**

We thank the reviewer for the comment. We agree with the suggestion and, therefore, have updated the study design to a retrospective cohort study.

Comment-2:

The study population or participants for the study is not clear; is it general TB patients or TB recurrence patients? Looking at the title of the study. This lack of clarity is seen in the analysis outputs.

**Response:**

Thank you so much for the observation. In this paper, we aim to report the epidemiology of TB recurrence in Tanzania. As such, we have reported the prevalence of TB recurrence, the factors associated with TB recurrence, and the factors associated with treatment outcomes of those with recurrent TB. As such, the analysis includes all patients and the sub-population of those with recurrent TB.

**Results**


Comments -Reviewer #1: Yes, the analysis was matched with the plan and the results were presented. Figure, table, and images are clearly stated

**Response**: We thank the reviewer for this comment.

Comments -Reviewer #2: The analysis presented, matches the intent and the analysis plan laid out by the authors. The results are appropriately written. The authors have tested and presented the results mainly in the form of tables, which is an acceptable way for such data.

**Response**: We thank the reviewer for this comment.

Comments - Reviewer #3: Wondering the choice of the study participants: The total TB patient in the data set (319,720) or the Patients with TB recurrence (6,310).
I thought the preliminary analysis (sociodemographic) should be focus on TB recurrence data set Not General TB dataset.  The total dataset is not relevant, and will not bring out the silent features of the main participants in the study, such as gender difference among the TB recurrence patients etc.

The result looked at the determinants which is part of the epidemiology of TB recurrence, and the distribution such as prevalence.

I think N in the title of table 2 is 6,310 instead of N=273,807. Likewise, in Table 4 with N there should be 519 NOT 4,884.

We thank the reviewer for the above comments.

To clarify, to achieve the objectives of the paper, we conducted two sets of analyses as follows:

1. We used the whole dataset (**N=319,720**) to determine the prevalence and factors associated with TB recurrence.
2. And use a sub population with recurrent TB (**N=5,419**) to determine the prevalence of unfavorable outcomes and associated factors among TB recurrent patients. -.

Since the initial analysis involves all TB patients, we included the socio-demographic characteristics of all TB patients in Table 1. However, we have computed bivariate analyses that focused only on patients with recurrent TB, as reported in supplementary table 2.

Table 2 shows the results of the multivariable analysis, reporting the factors that predispose all TB patients (N=273,807) to TB recurrence; hence, the total number of patients included in the analysis is 273,807. This number is different from the whole dataset sample of 319,720 because of the missing values in several independent variables.

Table 4 shows the results of the multivariable analysis, reporting the factors that predispose patients with TB recurrence (N=4,884) to unfavorable treatment outcomes; hence, the total number of patients included in the analysis is 4,884. This number is also different from the sample of 5,419 patients with recurrent TB because of the missing values in several independent variables.

--------------------

**Conclusions**


Comments - Reviewer #1: The conclusion is not separately stated in this research. it needs rewriting. The limitation of the analysis was clearly described. The authors described clearly the usefulness of the topic under the study. yes this work addressed the relevance of public health

**Response**: We thank the reviewer for this comment. We have included the conclusion in the last paragraph of the discussion according to PLOS NTD journal guidelines.

Comments - Reviewer #2: The conclusions are supported by the data presented. However, some of the recommendations presented after the data analysis can be toned down. For example: "We recommend post-treatment follow-up and prophylaxis with Isoniazid therapy or shorter regimen TB preventive therapy for patients with
recurrent TB, especially those coinfected with HIV."  How will this be achieved is beyond the scope of analysis and interpretation. This relies on so many factors and it questions the feasibility and involves socio-poltical structures in place, which is not appropriate for the conducted study, such recommendations should solely be based on results presented.

**Response**: Thank you so much for this comment. We have rephrased the stated recommendation to reflect our findings on page 25.

Comments - Reviewer #3: The conclusions are supported by the data with the limitations stated clearly and succinct discussion of public health importance.

**Response**: We thank the reviewer for this comment.

--------------------

**Editorial and Data Presentation Modifications?**

Comments - Reviewer #1: Minor revision is there in this study grammar correction, and the conclusion part total not written they need modification.

**Response**: We thank the reviewer for this comment. We have made grammatical corrections throughout the manuscript. The conclusion of the study is indicated as part of the discussion section.

Comments - Reviewer #2: The authors have relied on data presentation with just tables, which is appropriate; I wonder if some other reviewers felt a bit more graphical depiction of some of the key data would make it more appealing and easy for the readers.

**Response**: Thanks for this suggestion. We agree with your suggestion, however, given the extent of information being presented in this paper and the fact that we have presented one figure already, we kindly request that we keep the presentation of results as is.

Comment - Reviewer #3: Accept with minor revision

**Response**: Thank you for this comment.

--------------------

**Summary and General Comments**

Comments -Reviewer #1: This research was important in addressing information regarding awareness of TB in the study area also for globally. in section conclusion may need rewriting the other section is good.

**Response**: Thank you for this comment. The conclusions have been incorporated in the discussion ( the last paragraph) according to the journal guidelines.

Comments - Reviewer #2: The study is well conducted and well written. I have pointed out some areas of improvement but that should not prevent the editors from accepting the study for publication.

**Response**: Thank you for this comment. We have addressed the suggested comments.

Comments -Reviewer #3: Interesting, large secondary data which highlight recurrent TB determinants, but would have depicted the sociodemographic features of TB recurrent patients instead of general TB patients in which the data was drawn.

**Response**: Thank you for this comment.

--------------------
