# Supplementary material for: Epidemiology and treatment outcomes of recurrent tuberculosis in Tanzania from 2018 to 2021 using the National TB dataset
Source: PLoS Negl Trop Dis. 2024 Feb 15;18(2):e0011968. doi: 10.1371/journal.pntd.0011968 (PMC10901333; doi:10.1371/journal.pntd.0011968)
Supplement: Attachment — Submitted filename: Responses to reviewer_23012024.docx [file pntd.0011968.s004.docx]

**23^rd^ January 2024**

Dear Editor and Editorial Team,

PLOS Neglected Tropical Diseases

**RE: Submission of a revised manuscript for publication in PLOS Neglected Tropical Diseases Journal**

Thank you very much for the reviews and the opportunity to revise the manuscript. We have carefully revised the manuscript and will upload clean and tracked changed versions. In addition, we have attached a document with detailed responses to the comments and suggestions.

On behalf of all the authors of the above-noted manuscript, I confirm that this submission has not been published and is not being considered for publication elsewhere and that all the authors have agreed to the submission of this manuscript to PLOS Neglected Tropical Diseases Journal.

Sincerely,

Belinda J Njiro

(On behalf of all co-authors)

**Responses to the Editor’s comment**

**Comment**: After carefully reviewing the re-submitted manuscript, I would like to request just one tiny modification before considering it acceptable for publication.

As you have designed a study to investigate the prevalence of TB recurrence and associated factors, your study is a "cross-sectional study". As you have designed a study to investigate the prevalence of TB recurrence and associated factors, your study is a "cross-sectional study". Please modify this throughout the text.

**Response**: We thank the editor for this comment. We have revised the study design to a cross-sectional study throughout the manuscript.
